# Supplementary material for: Mental health dynamics of adolescents: A one-year longitudinal study in Harari, eastern Ethiopia
Source: PLoS One. 2024 Apr 18;19(4):e0300752. doi: 10.1371/journal.pone.0300752 (PMC11025968; doi:10.1371/journal.pone.0300752)
Supplement: Attachment — Submitted filename: PLOS review 1.docx [file pone.0300752.s002.docx]

Thank you for the opportunity to review this manuscript titled “Mental health dynamics of adolescents: A one-year longitudinal study in Harar, eastern Ethiopia”

The study objective is relevant and not often looked at in LMIC contexts.

**Introduction**

Re-write the introductory sentence *“Because of fast social, psychological, cognitive, and emotional changes, adolescence is a vulnerable period for mental illness and its effects”.*

The authors should also update their background with studies carried out in sub-Saharan context. There is a lot of relevant work that they could cite.

Paragraph three shares relevant information, but it seems out of place in context of the whole work. The authors should rewrite the introduction.

**Methods**

The methods section is clear.

The authors mention that the questions were read out to the adolescents. Can they let us know why they chose to use this method, and did they have to explain difficult concepts and how did they deal with this?

**Discussion**

In the 4^th^ paragraph, the introductory sentence reads “Our finding suggested that adolescent mental health problems were linked to an older age” and contradicts the following sentence. Correct that.

There is some answering of the study objectives, but the authors are making assumptions or conclusions that are not supported by the results. I suggest that they re-write this section.

**Conclusion**

“For middle-aged men, low-income men, and low-income women, school-based programs promoting prevention, early identification, and fast intervention are essential”. Re write this sentence as it looks like you are working with an adult population.
