# Supplementary material for: Mental health dynamics of adolescents: A one-year longitudinal study in Harari, eastern Ethiopia
Source: PLoS One. 2024 Apr 18;19(4):e0300752. doi: 10.1371/journal.pone.0300752 (PMC11025968; doi:10.1371/journal.pone.0300752)
Supplement: Attachment — Submitted filename: Response to Reviewrs.docx [file pone.0300752.s003.docx]

**February 10, 2023**

**To: PLOS ONE journal Editor-in-chief**

**Subject: Revision and resubmission of the manuscript (manuscript ID:** PONE-D-22-31044**)**

**Dear Editor and reviewers**,

We would like to express our appreciation to the editor and the reviewers for their insightful and constructive comments on our manuscript entitled ***“Mental health dynamics of adolescents: A one-year longitudinal study in Harar, eastern Ethiopia.”*** The comments and suggestions offered by the reviewers have immensely helped us to improve our manuscript.

We have considered every comment and have responded point by point, indicating how we addressed them and tracking the changes we have made. The changes are presented in the revised manuscript. In addition to our responses to the reviewers’ comments are provided in the table below here are suggested modification authors recommend. The number incorporated in the previous version "6724" is incorrectly stated in financial disclosure, and we kindly ask you to delete it from the online form.

The following amendment statement should be incorporated in online submission form on behalf of us.

“Haramaya University provided financial support for our study under the Scientific Research Grant number “HURG-2020-02-01-92”. The financial support was fifty thousand Ethiopian birr. The funders had no role in study design, data collection and analysis, decision to publish, or preparation of the manuscript.”

The data used for this study are also included and attached as supporting information.

We hope the revised manuscript is better suited to your esteemed journal and we are happy to consider any further revision as deemed necessary.

Sincerely,

Gari Hunduma

I. REPLY TO COMMENTS FROM EDITORS

| **Comments / suggestions** | **Response** |
| --- | --- |
| Please ensure that your manuscript meets PLOS ONE's style requirements, including those for file naming | Throughout the review, we verified the guidelines and adhered to all instructions and criteria. |
| Please provide additional details regarding participant consent. In the ethics statement in the Methods and online submission information, please ensure that you have specified  (1) whether consent was informed and  (2) What type you obtained (for instance, written or verbal, and if verbal, how it was documented and witnessed).  If your study included minors, state whether you obtained consent from parents or guardians.  If the need for consent was waived by the ethics committee, please include this information. | Your suggestion is appreciated. We added a statement about participant consent to the method section of our revised manuscript. We obtained parental or guardian consent for minors and consent from participants aged 18 and up because our study included minors. For minors, one participant's parents or legal guardians provided written, informed, and signed consent, and the adolescents provided written informed assent. Those over 18 were provided written, informed, and signed consent forms. We stated that written informed consent was obtained from parents or guardians for participants aged 13 to 17, and written informed consent was obtained from participants aged 18 and up. |
| We note that the grant information you provided in the ‘Funding Information’ and ‘Financial Disclosure’ sections do not match. When you resubmit, please ensure that you provide the correct grant numbers for the awards you received for your study in the ‘Funding Information’ section. Thank you for stating the following financial disclosure: "6724"  At this time, please address the following queries:   a) Please clarify the sources of funding (financial or material support) for your study. List the grants or organizations that supported your study, including funding received from your institution. b) State what role the funders took in the study. If the funders had no role in your study, please state: “The funders had no role in study design, data collection and analysis, decision to publish, or preparation of the manuscript.” c) If any authors received a salary from any of your funders, please state which authors and which funders. d) If you did not receive any funding for this study, please state: “The authors received no specific funding for this work.”   Please include your amended statements within your cover letter; we will change the online submission form on your behalf. | Thank you for your correction.  We apologize for the inconsistency of grant information in the 'Funding Information' and 'Financial Disclosure' sections.  The number **"6724"** is misspelled as financial disclosure, and we kindly ask you to delete it. Please replace the correct grant number with "HURG-2020-02-01-92.  Here in the revised manuscript, we clearly stated that **Haramaya University** provided financial and material support for our study under the Scientific Research Grant number (HURG-2020-02-01-92). The donation included fifty thousand Ethiopian birrs, stationery items such as duplication paper, printer-toner, pencils, and transportation to collect data. The funder has no role in study design, collection, analysis, the decision to publish, or the preparation of the manuscript. We have included our revised statements in our cover letter so you can change the online submission form.  “Haramaya University provided financial support for our study under the Scientific Research Grant number “HURG-2020-02-01-92”. The financial support was fifty thousand Ethiopian birr. The funders had no role in study design, data collection and analysis, decision to publish, or preparation of the manuscript.” |
| Upon re-submitting your revised manuscript, please upload your study’s minimal underlying data set as either Supporting Information files or to a stable, public repository and include the relevant URLs, DOIs, or accession numbers within your revised cover letter. | Thank you very much. We provided the data set as “supporting information files”. |
| I Agree with the first reviewer; the manuscript is difficult to follow due to extensive grammatical errors. Therefore, I suggest you rewrite the manuscript with the help of a native English speaker. | We appreciate your feedback. As suggested, language experts edited the English. The revised manuscript has improved its grammar and sentence structure. |
| **Reviewer #1 Evaluation** |  |
| The paper is a good start and a good area of interest. But a coherent flow in some areas is lacking, especially the introduction and discussion. I suggest that the authors re-write the manuscript with the help of a native English speaker. | Thank you and accepted as presented.  We appreciate your feedback. As suggested, language experts edited the English. The introduction and discussion are also rewritten and modified, as noted in the revised manuscript with track changes. With the assistance of a native English speaker, we rewrote the manuscript. Grammar and sentence structure have been improved in the revised manuscript. |
| **Introduction**  Re-write the introductory sentence “Because of fast social, psychological, cognitive, and emotional changes, adolescence is a vulnerable period for mental illness and its effects”.  The authors should also update their background with studies carried out in sub-Saharan context. There is a lot of relevant work that they could cite.  Paragraph three shares relevant information, but it seems out of place in context of the whole work. The authors should rewrite the introduction. | Thank you for your insightful comment.  As suggested, we re-wrote the introductory sentence as noted in the revised manuscript with track changes,  As noted in the revised manuscript with track changes, we also updated our background with studies conducted in the Sub-Saharan context. |
| **Methods**  The methods section is clear.  The authors mention that the questions were read out to the adolescents. Can they let us know why they chose to use this method, and did they have to explain difficult concepts and how did they deal with this? | Thank for your important point.  We used a guided, self-administered data collection technique to collect data from adolescent students at their schools. Each session had two data collectors whose main role was to facilitate, read the questions to the participants if necessary, and then allow them to choose acceptable responses based on the questionnaire frameworks.  We did this to ensure that participants understood the questionnaires and could discuss them with the data collectors if necessary in order to create a clear questionnaire. Those who assisted in data collection were asked to read the questions and explain them to the participants. |
| **Discussion**  In the 4^th^ paragraph, the introductory sentence reads “Our finding suggested that adolescent mental health problems were linked to an older age” and contradicts the following sentence. Correct that.  There is some answering of the study objectives, but the authors are making assumptions or conclusions that are not supported by the results. I suggest that they re-write this section. | Thank you for indicating this area.  We checked and corrected it in the revised manuscript and indicated it as highlighted.  We rewrote this section after reviewing our study objectives and conclusions, which were supported by the results. |
| **Conclusion**  “For middle-aged men, low-income men, and low-income women, school-based programs promoting prevention, early identification, and fast intervention are essential”. Re write this sentence as it looks like you are working with an adult population. | We appreciate your point. We rewrote this section after reviewing the conclusions, which were supported by the results. |
| **Reviewer #2 Evaluation** |  |
| The research is important work which is dire of literature in Africa. The work, though the findings are not statistical significant will serve as a pilot for future larger studies.  The authors need to make a few corrections i.e. line 34: 'McNemar's test' instead of 'McNamara's test'. line 69: The article you reference states ''Global prevalence of CMD in adolescents was 25.0% and 31.0%, using the GHQ cut-off point of 4 and 3, respectively.  '' The female and male figures are not in the paper. both figures are less then 25 so the sentence does not make sense. line 112:  Attrition 358 to 328 - Please provide possible reason and its effects on your result. | Thank you for your insightful comments.  In the revised manuscript, the correction is highlighted.  We included the following statements in the article: "Using the GHQ cut-off points of 4 and 3, respectively, the global prevalence of CMD in adolescents was 25.0% and 31.0%."  The male and female figures that were not included in the paper were removed.  The attrition rate and its possible cause were also described in the result section, as highlighted in the revised manuscript. |
