# Supplementary material for: Mental health dynamics of adolescents: A one-year longitudinal study in Harari, eastern Ethiopia
Source: PLoS One. 2024 Apr 18;19(4):e0300752. doi: 10.1371/journal.pone.0300752 (PMC11025968; doi:10.1371/journal.pone.0300752)
Supplement: Attachment — Submitted filename: Response to Reviewrs.docx [file pone.0300752.s004.docx]

**August 18, 2023**

**To: PLOS ONE journal Editor-in-chief**

**Subject: Revision and resubmission of the manuscript (manuscript ID: PONE-D-22-31044R1)**

**Dear Editor**,

We would like to express our appreciation to the editor and the reviewers for their insightful and constructive comments on our manuscript entitled ***“Mental health dynamics of adolescents: A one-year longitudinal study in Harar, eastern Ethiopia.”***

We have seen that the comments and suggestions offered by the editor have immensely helped us to improve our manuscript. We have considered every comment raised and have responded point by point, indicating how we addressed them and tracking the changes we have made. The changes are presented in the revised manuscript and also our responses to the comments are provided in the table below with suggested modification of the authors.

We hope the revised manuscript is better suited to your esteemed journal and we are happy to consider any further revision as deemed necessary.

Sincerely,

Gari Hunduma

REPLY TO COMMENTS RAISED FROM EDITORS.

| **Comments / suggestions** | **Response** |
| --- | --- |
| Your abstract is too lengthy; try summarizing it into 250 words or less. | Thank you for the suggestion, we edited the abstract and summarized it to the acceptable range of length. |
| Remove the statement ‘We assumed that the number of in-school adolescents with mental health problems would remain stable throughout the study’ from the abstract. | Your suggestion is appreciated. We remove the statement “We assumed that the number of in-school adolescents with mental health problems would remain stable throughout the study” from the abstract. |
| Can you please clarify what you meant by the statement below?  ‘To lessen adolescent mental health issues, quick interventions that emphasize middle age, the male gender, and wealth disparity are required.’  Kindly reword this statement …may be something like ‘interventions should focus on middle-aged and male adolescents from low-income families’ it sounds more reasonable. | Thank you for your correction.  We reworded the statements as “This highlighted that interventions for reducing adolescent mental health problems should focus on middle-aged and male adolescents from low-income families.” |
| Some parts of the manuscript contain punctuations before and after in-text citations, e.g., lines 82, 88, and 98; kindly edit these typos. Also, check the spacing between the in-text citations and the last words. In some, there is space between them, while in others none, e.g., lines 69, 81, etc. These are all over the manuscript. | Thank you very much. We edit these typos throughout the manuscript as indicated in the revised manuscript |
| Line 296 and in other parts of the manuscripts consider replacing ‘khat chewing, tobacco smoking, alcohol drinking’ with ‘the use of khat, tobacco, and alcohol.’ You can even say Khat use, tobacco use, and alcohol use. | We appreciate your feedback. As suggested, we replace the phrases as ‘Khat use, tobacco use, and alcohol use.’ In the revised manuscript. |
| End your introduction by explaining how your findings could benefit society, such as through policy changes or improving respondents' health. | Thank you for the suggestion. We end the introduction by stating “The finding may help to provide crucial data for policymakers in designing, planning, and implementing interventions of risk reduction and scale-up of services for adolescent mental health conditions.” |
| Last statement in your conclusion on page 22, line 348, remove tolerated. | Thank you for your correction again. The word ‘tolerated’ is removed from the revised manuscript and the statement is corrected as “Tailored interventions for reducing adolescent mental health problems should focus on middle-aged and male adolescents from low-income families.” |
| Reviewer #2:  Line 123 - 'Harare’s' SHOULD READ 'Harari's' | Thank you for the suggestion. We corrected it as suggested. |
