# Supplementary material for: Mental health dynamics of adolescents: A one-year longitudinal study in Harari, eastern Ethiopia
Source: PLoS One. 2024 Apr 18;19(4):e0300752. doi: 10.1371/journal.pone.0300752 (PMC11025968; doi:10.1371/journal.pone.0300752)
Supplement: Attachment — Submitted filename: Response to Reviewrs.docx [file pone.0300752.s005.docx]

**Author's Responses to Questions or Responses to reviewers**

Review Comments to the Author

Reviewer # 4:

All comments have been addressed. The manuscript has been revised accordingly, and now from my side it is acceptable. The statistical part should be revised by an expert

**Response**

Thank you for your positive feedback and accepted as presented.

To provide a detailed explanation of the statistical methods used in our study, we start by describing the purpose of each test and how it was applied to our data. **Wilcoxon matched-pairs signed-rank test**: This test is used to compare two related samples, such as before-and-after measurements or matched pairs of subjects. It tests whether the median difference between the two samples is significantly different from zero. In our study, this test was used to compare the median score severity charges of mental health problems among different groups of study participants as depicted in table 4.

- **We used McNemar’s Chi-squared test** to compare two paired proportions, such as the proportion of subjects who respond positively during the base line assessment and during the follow up assessment. It tests whether the proportion of subjects who change from one category to another is significantly different from what would be expected by chance. We used this test to compare the severity charges of mental health problems among different groups of study participants (table 5).
- **Random-effects logistic regressions on panel data**: This method is used to analyze longitudinal data, where the same subjects are measured repeatedly over time. It models the relationship between a binary outcome variable (such as the presence or absence of a mental health disorder) and one or more predictor variables (such as age, gender, or treatment group). In our study, this method was used to find the potential factors associated with individual mental health disorders (table 6).

Review Comments to the Author

Reviewer #5:

1. This is a good paper. However, it needs some improvements.

Response: Thank you for your positive feedback.we try to address the raised issues points by point and we hope that we improved our revised manuscript.

2. The abstract should be re-write after attending to recommendations.

**Response:** We appreciate your point. The abstract makes this clear under the conclusion part. We re-write the paragraph in this way. “The prevalence of mental health problems were high among the study cohort. The proportion of overall problems and externalizing problems has increased over time, indicating a **deterioration** in the mental health of the study cohort. However, the decrease in internalizing problems among older adolescents, girls, and those with an average wealth index is a positive sign. The findings highlight that tailored interventions are required to reduce externalizing problems and maintain the decrease in internalizing problems. These interventions should **target middle-aged and male adolescents from low-income families”.**

3. Authors wrote a very good introduction. However, there is a need to write done hypothesis as authors wanted to test whether there were differences in SDQ score of T1 and T2.

**Response:** We are grateful and the comment is acceptable. We hypothesized that the number of in-school adolescents with mental health problems would remain stable throughout the study, and therefore there would be no significant difference between the SDQ score of T1 and T2.this is included in the revised manuscript. Please kindly refer lines 104-106.

4. **Methods:** -Study setting, designs, and samples: Line 107: how many schools exist in the region and how authors conducted the random selection? Were they choosing one per cluster, or they listed all schools and use MS Excel or any software for random selection... Same for selection of participants (line 117).

**Response:** Thank you for raising this point. During the data collection period, seven governmental high schools were in the region. We used a simple random sampling technique to select the schools. We listed all schools and randomly selected three schools using computer-generated sampling. We identified grade levels for each selected school, and finally, sections were selected proportionally using the lottery method from each grade level, considering the number of sections. Finally, participants were selected randomly from each section using systematic random sampling. All are incorporated in the revised manuscript.

5. **Data collection tool:** How did authors do to identify participants after one year for T2 data collection and reconcile with T1 data for the sample selection was dependent T1 and T2: did they use identifiable such names, or ID...

**Response:** Thank you for bringing up such crucial points. In order to ensure that the data collected from participants at different times could be linked, we used a unique ID code to identify participants for T2 data collection after a year. This allowed us to reconcile the data with T1 data analysis. It is included in the revised manuscript.

6. Line 130 and 131: authors state that "...we conducted pretests and confirmed Cronbach's alpha for reliability and validity..." Was this conducted as a pilot study? They included how many participants in the pilot study?

**Response:** Thank you for your feedback. We conducted pretests on 5% of the sample size to confirm the reliability and validity of the items. All data collection instruments were pre-tested in Dire Dawa administrative counselling among similar in-school adolescents in 5% of the sample size. However, we did not conduct this as a pilot study. We calculated Cronbach’s alpha for the reliability and validity of the tool to assess the scales’ internal consistency and reliability before actual data collection. We apologize for any confusion this may have caused. And it is incorporated the revised manuscript as shown in track change.

7. Variables and Measurements: some variables are in the results section in table 1 but authors did not define them in the methods section e.i. Wealth index, Alcohol use, FCV-19S...

**Response:** We appreciated your comments. All important variables in the results section are defined in the methods section including Wealth index, substance use, such as alcohol, cigarettes, khat, or other illicit drugs, Bullying at school and Self-esteem:as per the suggestions.

8. Regression models are parametric statistic tests. This means that to feed variables in the model, one needs to find out, after univariate analysis, about the homoscedasticity and lineality of these variables, not only about a p value of <0.1 or 0.2.

**Response:** Thank you for your feedback. We have checked the assumptions of linearity, independence, homoscedasticity, and normality of the model during our data analysis. We performed univariate analysis to check for the homoscedasticity and linearity of the variables before feeding them into the model. We also checked for normality by examining the distribution of the residuals. We ensured that these assumptions were met before feeding the variables into the regression model and it is incorporated in the revised manuscript. Thank you for bringing this to my attention.

9. Authors repeatedly assess Cronbach alpha while this was not part of the study objectives. Yet they did not even define how they interpreted it in the methods section.

**Response:** Thank you for your feedback. We acknowledge that Cronbach’s alpha was assessed repeatedly in our study. We will ensure that we only assess Cronbach’s alpha if it is relevant to the study objectives in future studies. Additionally, we defined how Cronbach’s alpha was interpreted in the methods section of the study to help readers understand the rationale behind the assessment of Cronbach’s alpha. It is described as “Cronbach’s alpha is a measure of the internal consistency or reliability of a set of survey items. It quantifies the level of agreement on a standardized 0 to 1 scale, with higher values indicating higher agreement between items. Cronbach’s alpha is used to determine whether a collection of items consistently measures the same characteristic. In this study, we assessed Cronbach’s alpha to evaluate the internal consistency of our survey items” in the revised manuscript.

10. Results -Table 3: It could be better if authors calculated the weighted rate.

**Response:** Thank you for your feedback. Our intention was to show the prevalence distributions of self-reported adolescents’ mental health problem symptoms with ages and the corresponding time of the survey (T1 & T2). We agree that calculating the weighted rate could be useful in adjusting for differences in the distribution of confounding variables between the study groups. However, we believe that our study groups were similar in terms of these variables, and therefore, calculating the weighted rate may not be necessary. We hope this explanation helps clarify our reasoning for not calculating the weighted rate.”
